# Comparison of radiographic and clinical outcomes between ALIF, OLIF, and TLIF over 2-year follow-up: a comparative study

**DOI:** 10.1186/s13018-023-03652-5

**Published:** 2023-03-02

**Authors:** Kuan-Kai Tung, Wei-Cheng Tseng, Yun-Che Wu, Kun-Hui Chen, Chien-Chou Pan, Wen-Xian Lu, Cheng-Min Shih, Cheng-Hung Lee

**Affiliations:** 1 Department of Orthopedics, Taichung Veterans General Hospital, Taichung, Taiwan; 2 Department of Biomedical Engineering, Hung Kuang University, Taichung, Taiwan; 3 Department of Computer Science and Information Engineering, Providence University, Taichung, Taiwan; 4 Department of Nursing, Jenteh Junior College of Medicine, Nursing and Management, Miaoli, Taiwan; 5 Department of Rehabilitation Science, Jenteh Junior College of Medicine, Nursing and Management, Miaoli, Taiwan; 6 Department of Orthopedics, Feng Yuan Hospital Ministry of Health and Welfare, Taichung, Taiwan; 7 Department of Physical Therapy, Hung Kuang University, Taichung, Taiwan; 8 Post Baccalaureate Medicine, School of Medicine, National Chung Hsing University, Taichung, Taiwan; 9 Department of Food Science and Technology, Hung Kuang University, Taichung, Taiwan

**Keywords:** Degenerative spine disorders, Lumbar interbody fusion, ALIF, OLIF, TLIF, Clinical outcome

## Abstract

**Background:**

Regarding the increasing adoption of oblique lateral interbody fusion (OLIF) for treating degenerative lumbar disorders, we aimed to evaluate whether OLIF, one of the options for anterolateral approach lumbar interbody fusion, demonstrate clinical superiority over anterior lumbar interbody fusion (ALIF) or posterior approach, represented by transforaminal lumbar interbody fusion (TLIF).

**Methods:**

Patients who received ALIF, OLIF, and TLIF for symptomatic degenerative lumbar disorders during the period 2017–2019 were identified. Radiographic, perioperative, and clinical outcomes were recorded and compared during 2-year follow-up.

**Results:**

A total of 348 patients with 501 correction levels were enrolled in the study. Fundamental sagittal alignment profiles were substantially improved at 2-year follow-up, particularly in the anterolateral approach (A/OLIF) group. The Oswestry disability index (ODI) and EuroQol-5 dimension (EQ-5D) in the ALIF group were superior when compared to the OLIF and TLIF group 2-year following surgery. However, comparisons of VAS-Total, VAS-Back, and VAS-Leg revealed no statistically significance across all approaches. TLIF demonstrated highest subsidence rate of 16%, while OLIF had least blood loss and was suitable for high body mass index patients.

**Conclusions:**

Regarding treatment for degenerative lumbar disorders, ALIF of anterolateral approach demonstrated superb alignment correction and clinical outcome. Comparing to TLIF, OLIF possessed advantage in reducing blood loss, restoring sagittal profiles and the accessibility at all lumbar level while simultaneously achieving comparable clinical improvement. Patient selection in accordance with baseline conditions, and surgeon preference both remain crucial issues circumventing surgical approach strategy.

## Introduction

Degenerative spine pathology, including isthmic spondylolisthesis, lumbar spinal stenosis, and degenerative disk disease, may accompany the clinical deterioration of neurologic deficits which being negatively related to a health-related quality of life (HRQoL) [1]. Recently, the increased demand for patients to return to work as early as possible and the avoidance of postoperative complications have led to the development of techniques offering a faster recovery period. Lumbar interbody fusion (LIF), including posterior lumbar interbody fusion (PLIF), transforaminal lumbar interbody fusion (TLIF), anterior lumbar interbody fusion (ALIF), lateral lumbar interbody fusion (LLIF), and oblique lumbar interbody fusion (OLIF), are able to provide good to excellent clinical results for the treatment of degenerative spine conditions in a minimally invasive assessment [2–5].

In the current study, we adopted the OLIF procedure rather than the transpsoas LLIF procedure, since OLIF allows for a similar sized cage to be placed while avoiding irritation to the psoas muscle through accessing the anatomical corridor anterior to the psoas muscle. In the sagittal plane, how significant a role the OLIF plays in alignments restoration and clinical improvement have been frequently debated. However, previous reports have only involved a relatively small study size and high heterogeneity in surgical indications, a short follow-up period, and clinical outcome reports. Discussions circumventing OLIF procedures remain scarce despite the recent increase in the adoption of this technique.

The current study aimed to determine if the OLIF procedure demonstrates clinical superiority over ALIF or TLIF in degenerative spine pathologies in long-term follow-up. In addition, the outcomes of the anterolateral approach (A/OLIF) group and posterior approach (TLIF) group were compared in a subanalysis. Both radiographic and clinical outcomes as well as patient-reported HRQoL were fully reported in this study.

## Materials and methods

### Data source and study population

The study population for this research consisted of patients who underwent lumbar interbody fusion, including ALIF, OLIF, and TLIF, for symptomatic degenerative lumbar disorders between 2017 and 2019 at our hospital. The inclusion criteria for the study subjects were: (1) patients presenting with lower back pain or sciatica that did not respond to conservative treatment for over 6 months due to degenerative spinal conditions; (2) lumbar interbody fusion with no more than 4 index levels fused; (3) complete follow-up records; and (4) complete HRQoL assessment at 2-year follow-up. Patients who were followed up for less than 2 years, had malignancy, neuromuscular disease, or spinal fractures were excluded from the study. Demographic and clinical data, including age, body mass index (BMI), gender, surgical technique, and hospitalization length of stay (HLoS), as well as intraoperative factors such as estimated blood loss (EBL) and operative duration (OPD) were all assessed.

### Radiographic outcome-sagittal parameters

Full-length lateral spine radiographs of the kyphosis series (36 inch) were taken during pre-OP visits, at 6 months after surgery and at 2-year follow-up period. Radiographs were analyzed for global alignment parameters by K-K T and W-C T using validated Surgimap surgical planning software (Nemaris Inc., New York, NY, United States). Radiographic measurements were performed while patient was positioned at a central location based upon standardized techniques, including the lumbar lordosis (LL, the lordotic angle from the superior endplate of L1 to the superior endplate of S1), pelvic incidence (PI, defined as the angle subtended by a line drawn perpendicular to the superior endplate of S1 and a line drawn from the center of the femoral head to the midpoint of the superior endplate of S1), PI minus LL, pelvic tilt (PT, defined as the angle made between lines originating at the bicoxofemoral axis and extending vertically to the middle of the superior endplate of S1), sacral slope (SS) and sagittal vertical axis (SVA, defined as the distance from the posterosuperior corner of the S1 body to the C7 plumb line) [6].

### Radiographic outcome-fusion and subsidence

**Fig. 1:**
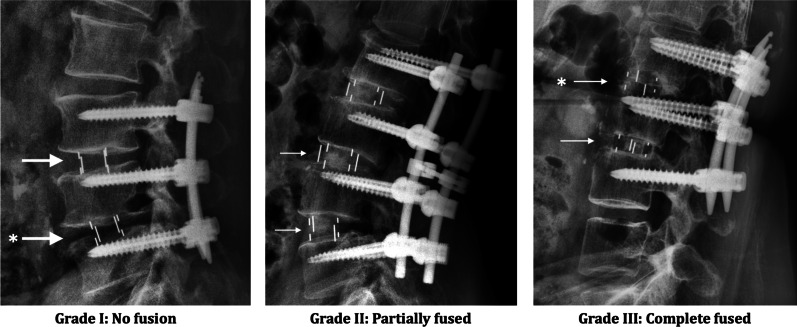
Grading for fusion status. Arrow ( →), interbody cage in position. Asterisk (*), the subsidence of LIF cage. Noted that cage subsidence can be observed in different fusion status

K-K T and W-C T reviewed the flexion–extension films of the lumbar and recorded their fusion status. Each fusion level was evaluated independently using the Hutter method [7] according to the Santos criteria [8, 9] of fusion grading at 2-year follow-up (Fig. 1): (1) Grade I: No fusion. Any motion or radiolucency around the device; (2) Grade II: Partially fused. No motion around the device without definite bony opacity formation in/around the cage; (3) Grade III: Completely fused: No motion or radiolucency around the device with definite bony opacity formation in/around the cage. A successful fusion was defined by Grade II and/or Grade III among the study cohorts. Interbody cage subsidence was defined as a sinking of the interbody cage due to progressive endplate collapse ≧ 2 mm at the follow-up time period. The coexistence of cage subsidence and bony fusion is possible.

### Clinical outcomes

Clinical outcomes were evaluated using standardized self-reported measures of health-related quality of life (HRQoL), including the Oswestry disability index (ODI), the EuroQol-5-dimension score (EQ-5D), the visual analogue scale of pain for total symptoms (VAS-Total), for symptoms in the affected leg (VAS-Leg), and for symptoms in the back (VAS-Back), at 1 month, 3 months, 6 months, 1 year, and 2 years after surgery. A clinically relevant successful treatment for lumbar interbody fusion surgery was defined as one that achieved a predetermined cutoff value for minimal clinically important difference (MCID). A reduction of 2 in VAS-Total, 20 in ODI, 2.5 in VAS-Back, 3.5 in VAS-Leg, and 0.3 in EQ-5D were considered to be acceptable MCID values for the individual [10, 11]. The proportion of patients who achieved MCID was measured in all study groups.

### Statistical analysis

All quantitative variables were reported as the mean and standard deviation, and qualitative variables were presented as ratios and numbers. Continuous variables were analyzed using the paired Student's t test and one-way analysis of variance (ANOVA). Categorical variables were analyzed using the Pearson Chi-square test and the Mann–Whitney test. When statistical significance was reached between the groups, a post hoc analysis (using Turkey’s method and Bonferroni correction) was conducted. A *p*-value less than 0.05 was considered to indicate a statistically significant difference. We conducted a power analysis using the F test for a 3-group one-way ANOVA, with the parameters of alpha error = 5% and study power (1-beta) = 0.8. The total estimated sample size was 159, which is approximately 53 participants in each group.

## Results

**Fig. 2:**
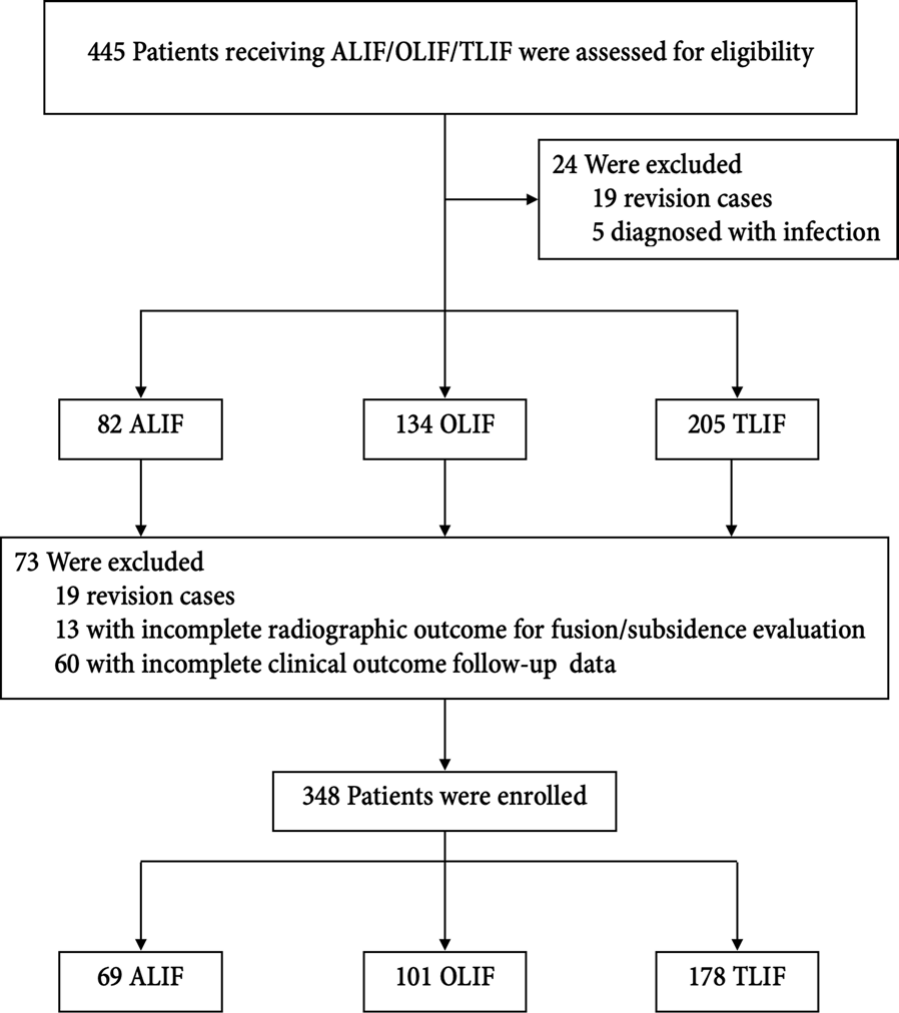
Numbers of patients who were screened and included in the study. *ALIF* Anterior lumbar interbody fusion; *OLIF* Oblique lumbar interbody fusion*; TLIF* Transforaminal lumbar interbody fusion

This study included a total of 445 eligible patients between January 2017 and December 2019, out of which 348 met the inclusion criteria. Among these 348 patients, 69 received anterior lumbar interbody fusion (ALIF), 101 received oblique lumbar interbody fusion (OLIF), and 178 received transforaminal lumbar interbody fusion (TLIF) at 84, 163, and 254 levels with posterior instrumentation, respectively, for the treatment of degenerative lumbar spine pathologies. (Fig. 2).

### Demographic and operative clinical data

Table 1 presents the demographic and clinical data for the patients in the ALIF, OLIF, and TLIF groups. The patient population consisted of 227 (65.2%) females, with a mean age of 63 ± 12 years. The ALIF group had significantly younger patients (55.3 ± 14.8 years, *p* < 0.01 while comparing to O/TLIF), while the OLIF group had a generally higher body mass index (BMI) (26.7 ± 4.3, *p* = 0.01 while comparing to A/TLIF). The estimated blood loss (EBL) was significantly lower in the OLIF group (410.4 ± 233.7 mL, *p* = 0.04 while comparing to A/TLIF). The most common lumbar degenerative pathologies were spinal stenosis (58.3%) and spondylolisthesis (64.1%). Most patients (65.2%) received single-level lumbar interbody fusion surgery, targeting the L5/S1 in the ALIF group (45.2%), and the L4/5 in the OLIF (54%) and TLIF (51.6%) groups. The overall fusion rate (grades II and III) was 94.6%, with 92.9%, 95.1%, and 94.9% in the ALIF, OLIF, and TLIF groups, respectively. The overall subsidence rate was 12.6%, with 7.1%, 9.8%, and 16.1% in the ALIF, OLIF, and TLIF groups, respectively. The TLIF group had a significantly higher subsidence rate, although the fusion rate was similar to the ALIF and OLIF groups. The mean correction levels, gender distribution, length of hospitalization, and outpatient visits were comparable between the three groups. The anterolateral approach group (A/OLIF) had a younger age (60.7 ± 12.3 vs. 65.2 ± 11.4 years, *p* < 0.01), a higher BMI (26.7 ± 4.3 vs. 25.4 ± 3.3 kg/m^2^, *p* < 0.01), and a relatively lower subsidence rate (8.9% vs. 16.1%, *p* = 0.03) compared to the TLIF group (Table 2). All study subjects were successfully discharged and followed up for two years after surgery without experiencing any spinal nerve, major vessel, peritoneal or urinary injuries after surgery.

**Table 1:** Demographic and perioperative data of the study population stratified by lumbar interbody fusion types

Characteristic	Overall (*n* = 348)	Group			
		ALIF (*n* = 69)	OLIF (*n* = 101)	TLIF (*n* = 178)	*p* value
Correction levels	501	84	163	254	
Mean correction levels	1.4	1.2	1.6	1.4	
Age—year (95% CI)	63 ± 12	55.3 ± 14.8	64.4 ± 8.6	65.2 ± 11.4	< 0.01**
	(61.8–64.3)	(51.7–58.8)	(62.7–66.1)	(63.6–66.9)	
Female gender—no. (%)	227/348	47/69	68/101	112/178	0.65
	(65.2)	(68.1)	(67.3)	(62.9)	
BMI—Kg/m^2^	26.1 ± 3.9	25.7 ± 3.6	26.7 ± 4.3	25.4 ± 3.3	0.01*
	(25.7–26.5)	(24.8–26.5)	(26.1–27.4)	(24.7–26)	
HLoS—days (95% CI)	7.9 ± 4	8.5 ± 4.5	8 ± 3.8	7.7 ± 3.9	0.38
	(7.5–8.3)	(7.4–9.6)	(7.2–8.7)	(7.1–8.3)	
OPD—min (95% CI)	362.4 ± 86.3	362.4 ± 103.4	370 ± 85	358.1 ± 79.8	0.54
	(353.3–371.5)	(337.6–387.2)	(353.2–386.8)	(346.3–369.9)	
EBL—mL (95% CI)	457.3 ± 421.1	548.6 ± 718.6	410.4 ± 233.7	519.4 ± 294.7	0.04*
	(412.9–501.7)	(375.9–721.2)	(364.2–456.5)	(475.8–563)	
*Pre-OP diagnosis—no. (%)*					
Discogenic pain	15 (4.3)	11 (11.8)	4 (3.1)	0	
HIVD	50 (14.4)	7 (7.5)	7 (5.5)	36 (12.8)	
Spinal stenosis	203 (58.3)	21 (22.6)	38 (29.9)	144 (51.1)	
Spondylolisthesis	223 (64.1)	49 (52.7)	78 (61.4)	96 (34)	
Spondylolysis	11 (3.2)	5 (5.4)	0	6 (2.1)	
*Operation level(s)—no. (%)*					
1 level	227 (65.2)	55 (79.7)	57 (56.4)	115 (64.6)	
2 levels	89 (25.6)	13 (18.8)	26 (25.7)	50 (28.1)	
3 levels	32 (9.2)	1 (1.4)	18 (17.8)	13 (7.3)	
*Index fusion level—no. (%)*					
L1/L2	3 (0.6)	0	2 (1.2)	1 (0.4)	
L2/L3	45 (9)	5 (6)	17 (10.4)	23 (9.1)	
L3/L4	112 (22.4)	11 (13.1)	50 (30.7)	51 (20.1)	
L4/L5	249 (49.7)	30 (35.7)	88 (54)	131 (51.6)	
L5/S1	92 (18.4)	38 (45.2)	6 (3.7)	48 (18.9)	
*Fusion status—no. (%)*					
Grade I	27	6	8	13	
Grade II	186	30	60	96	
Grade III	274	42	87	145	
Fusion rate—no. (%) (Grade II and III)	474/501	78/84	155/163	241/254	0.8
	(94.6)	(92.9)	(95.1)	(94.9)	
Subsidence—no. (%)	63/501	6/84	16/163	41/254	0.04*
	(12.6)	(7.1)	(9.8)	(16.1)	

*p* value < 0.05 was consider statistically significant. Values expressed as the mean ± standard deviation and 95 confidence interval range in the brackets. *p* < 0.05*, *p* < 0.01**

*ALIF* Anterior lumbar interbody fusion, *OLIF* Oblique lumbar interbody fusion, *TLIF* Transforaminal lumbar interbody fusion, *HLoS* Hospital length of stay, *EBL* Estimated blood loss, *BMI* Body mass index, *OPD* Operative duration, *HIVD* Herniated intervertebral disk disease

**Table 2:** Comparison of demographic and perioperative data between anterolateral and posterior approach

Characteristic	Group		*p* value
	Anterolateral (A/OLIF, *n* = 170)	Posterior (TLIF, *n* = 178)	
Correction levels	247	254	
Age-year	60.7 ± 12.3	65.2 ± 11.4	< 0.01**
	(58.8–62.6)	(63.6–66.9)	
Female gender (%)	68/101	112/178	0.65
	(67.3)	(62.9)	
BMI-Kg/m^2^	26.7 ± 4.3	25.4 ± 3.3	< 0.01**
	(25–26)	(24.7–26)	
HLoS-days	8.2 ± 4.1	7.7 ± 3.9	0.26
	(7.5–8.8)	(7.1–8.3)	
OPD—min (95% CI)	366.9 ± 92.7	358.1 ± 79.8	0.34
	(352.9–380.9)	(346.3–369.9)	
EBL– mL (95% CI)	466.5 ± 494.7	519.4 ± 294.7	0.22
	(391.6–541.4)	(475.8–563)	
Fusion rate—no. (%) (Grade II and III)	233/247	241/254	0.57
	(94.3)	(94.9)	
Subsidence—no. (%)	22/247	41/254	0.03*
	(8.9)	(16.1)	

*p* value < 0.05 was consider statistically significant. Values expressed as the mean ± standard deviation and 95 confidence interval range in the brackets. *p* < 0.05*, *p* < 0.01**

*ALIF* Anterior lumbar interbody fusion, *OLIF* Oblique lumbar interbody fusion, *TLIF* Transforaminal lumbar interbody fusion, *HLoS* Hospital length of stay, *EBL* Estimated blood loss, *BMI* Body mass index, *OPD* Operative duration

### Radiographic outcome

The sagittal alignment profiles, including PT, SVA, LL, SL, SS, and PI-LL mismatch, were substantially improved at the 2-year follow-up among the study cohort, particularly in the ALIF and OLIF groups (Table 3). The PI-LL mismatch and SVA of the ALIF and OLIF group were significantly superior to those seen in the TLIF group from the 6-month follow-up. Furthermore, SS, LL, and SL reached statistical significance when comparing them to the TLIF group 2 years after surgery.

**Table 3:** Radiographical outcome of the study population stratified by lumbar interbody fusion types

Characteristic	Overall (*n* = 348)	Group			*p* value
		ALIF (*n* = 69)	OLIF (*n* = 101)	TLIF (*n* = 178)	
*PT*					
Pre-OP	20.6 ± 7.1	20.8 ± 6.8	19.8 ± 8.2	21.1 ± 6.5	0.37
	(19.9–21.4)	(19.1–22.4)	(18.2–21.4)	(20.1–22)	
6 M	17 ± 4.5	16.9 ± 6.1	17.1 ± 3.6	17.1 ± 4.3	0.94
	(16.5–17.5)	(15.3–18.4)	(16.4–17.8)	(16.4–17.7)	
2 Y	16.5 ± 6.5	16.9 ± 8.1	16.9 ± 7.3	16.2 ± 5.2	0.66
	(15.8–17.2)	(14.9–18.8)	(15.4–18.3)	(15.4–17)	
*p* value (2 Y-pre)	< 0.01**	< 0.01**	< 0.01**	< 0.01**	
*SS*					
Pre-OP	32.9 ± 10.6	31.9 ± 13.1	34 ± 10.2	32.6 ± 9.8	0.38
	(31.7–34)	(28.7–35)	(32–36)	(31.1–34)	
6 M	34.7 ± 4.2	35.2 ± 6	34.5 ± 3.6	34.5 ± 3.6	0.53
	(34.2–35.1)	(33.7–36.7)	(33.8–35.3)	(34–35.1)	
2 Y	34.7 ± 6.8	37.2 ± 10.7	34.8 ± 6.5	33.7 ± 4.5	< 0.01**
	(34–35.4)	(34.6–39.7)	(33.5–36.1)	(33–34.4)	
*p* value (2 Y-pre)	< 0.01**	< 0.01**	0.48	0.18	
*PI*					
Pre-OP	53.5 ± 9	52.6 ± 11.5	53.7 ± 9	53.6 ± 7.9	0.66
	(52.5–54.4)	(49.8–55.3)	(52–55.5)	(52.5–54.8)	
6 M	53.9 ± 7	52.7 ± 8.4	53.7 ± 8.2	53.9 ± 5.2	0.57
	(52.1–53.6)	(51.6–55.8)	(52.3–55.6)	(52–53.7)	
2 Y	52.2 ± 7.6	53.8 ± 12	52.7 ± 8.2	52.0 ± 4	0.14
	(51.4–53)	(51–56.7)	(51–54.3)	(49.3–53.5)	
*p* value (2 Y-pre)	0.45	0.18	0.1	0.24	
*LL*					
Pre-OP	41 ± 11.3	39.2 ± 10.5	42.8 ± 12.3	40.7 ± 11	0.11
	(39.8–42.2)	(36.7–41.8)	(40.4–45.2)	(39–42.3)	
6 M	44.9 ± 8	45.6 ± 5.3	46 ± 3.1	44.1 ± 10.5	0.12
	(44.1–45.8)	(44.3–46.8)	(45.4–46.6)	(42.5–45.6)	
2 Y	42.8 ± 10.5	45 ± 17.4	44.9 ± 7.9	40.8 ± 7.5	0.01*
	(41.7–43.9)	(40.8–49.2)	(43.3–46.5)	(39.6–41.9)	
*p* value (2 Y-pre)	0.04*	< 0.01**	0.04*	0.42	
*SL*					
Pre-OP	17.2 ± 5.5	17.6 ± 5.1	16.2 ± 5.7	17.4 ± 5.6	0.37
	(16.6–17.8)	(16.4–18.8)	(15.1–17.3)	(16.6–18.2)	
6 M	20.5 ± 6.4	21.7 ± 6.4	20.6 ± 6	20.3 ± 7.4	0.07
	(19.8–21.2)	(20.2–23.2)	(19.4–21.8)	(19.2–21.4)	
2 Y	19.4 ± 8.5	21.4 ± 9.2	19.3 ± 7.1	19.1 ± 8.4	0.05*
	(18.5–20.3)	(19.9–23.6)	(17.9–20.7)	(17.7–20.5)	
*p* value (2 Y-pre)	0.04*	0.03*	0.03*	0.05*	
*SVA*					
Pre-OP	56.1 ± 30.7	55.5 ± 25.9	61.8 ± 37	51.2 ± 26.6	0.2
	(51–61.2)	(46.7–64.4)	(51.4–72.3)	(44.1–58.4)	
6 M	45.7 ± 14.7	47.6 ± 9.7	49.4 ± 8.9	43.4 ± 17.4	0.02*
	(43.9–47.5)	(44.5–50.6)	(47.3–51.5)	(40.6–56.2)	
2 Y	45 ± 42.6	45 ± 39.5	45.8 ± 43.6	54.5 ± 56.2	0.05*
	(42–48)	(41.3–49.8)	(40.2–51.4)	(46–63)	
*p* value (2 Y-pre)	0.03*	0.04*	0.03*	0.09	
*PI-LL*					
Pre-OP	14.6 ± 11.4	14.2 ± 10.6	13.3 ± 11.2	15.4 ± 11.8	0.33
	(13.4–15.8)	(11.6–16.7)	(11.1–15.5)	(13.7–17.2)	
6 M	7.5 ± 5.2	9.2 ± 5.5	7.8 ± 4	7.2 ± 5.6	0.04*
	(6.9–8)	(7.9–10.6)	(7–8.6)	(6.3–8)	
2 Y	11.9 ± 7.2	11.8 ± 8.5	10 ± 7.3	11.6 ± 6.3	0.04*
	(11.1–12.6)	(9.7–13.8)	(8.5–11.4)	(10.7–12.5)	
*p* value (2 Y-pre)	0.04*	0.03*	0.04*	0.06	

*p* value < 0.05 was consider statistically significant. Values expressed as the mean ± standard deviation and 95 confidence interval range in the brackets. *p* < 0.05*, *p* < 0.01**

*ALIF* Anterior lumbar interbody fusion, *OLIF* Oblique lumbar interbody fusion, *TLIF* Transforaminal lumbar interbody fusion, *PT* Pelvic tilt, *SS* Sacral slope, *PI* Pelvic incidence, *LL* Lumbar lordosis, *SL* Segmental lordosis, *SVA* Sagittal vertical axis, *PI-LL* PI minus LL mismatch, *M* Month, *Y* Year

### Clinical outcome

**Fig. 3:**
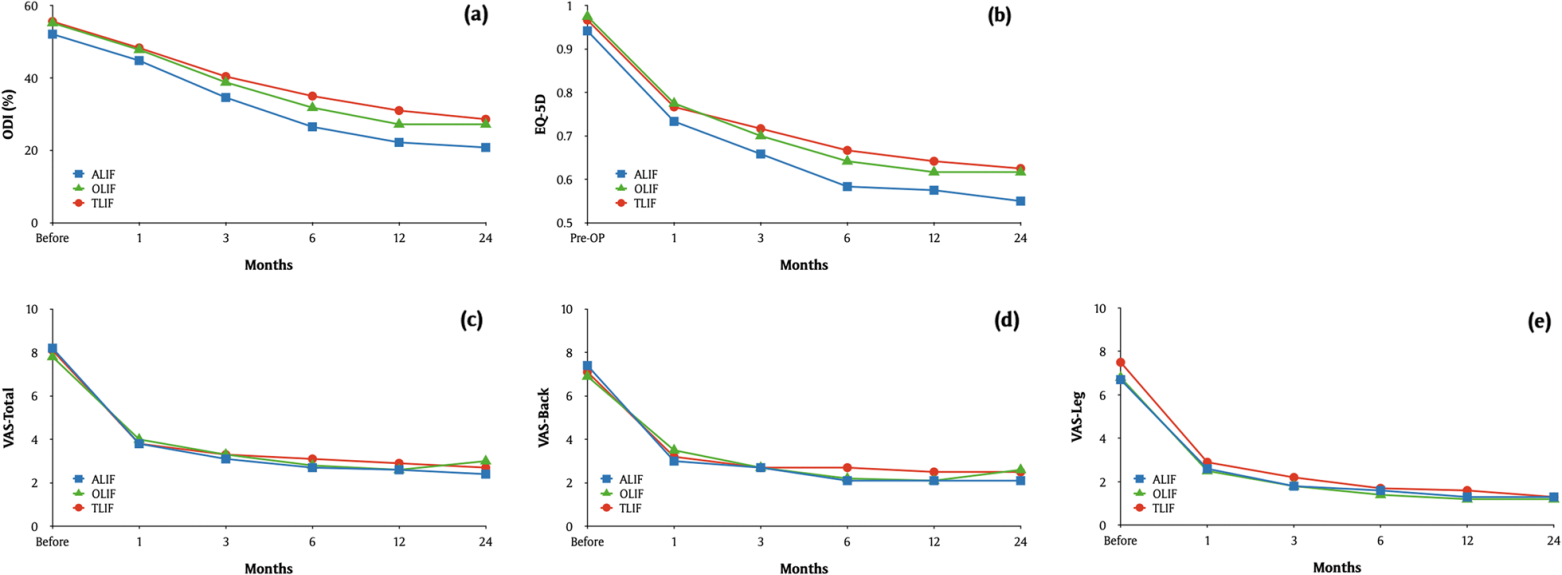
Comparison of clinical outcome between ALIF, OLIF, and TLIF over 2 years. **a** ODI **b** EQ-5D **c** VAS-Total **d** VAS-Back **e** VAS-Leg. *ALIF* Anterior lumbar interbody fusion; *OLIF* Oblique lumbar interbody fusion; *TLIF* Transforaminal lumbar interbody fusion; *ODI* Oswestry disability index; *EQ-5D* EuroQol-5-dimension score; *VAS-Total* VAS of Pain in Total; *VAS-Leg* VAS of pain in leg; *VAS-Back* VAS of pain in back

**Fig. 4:**
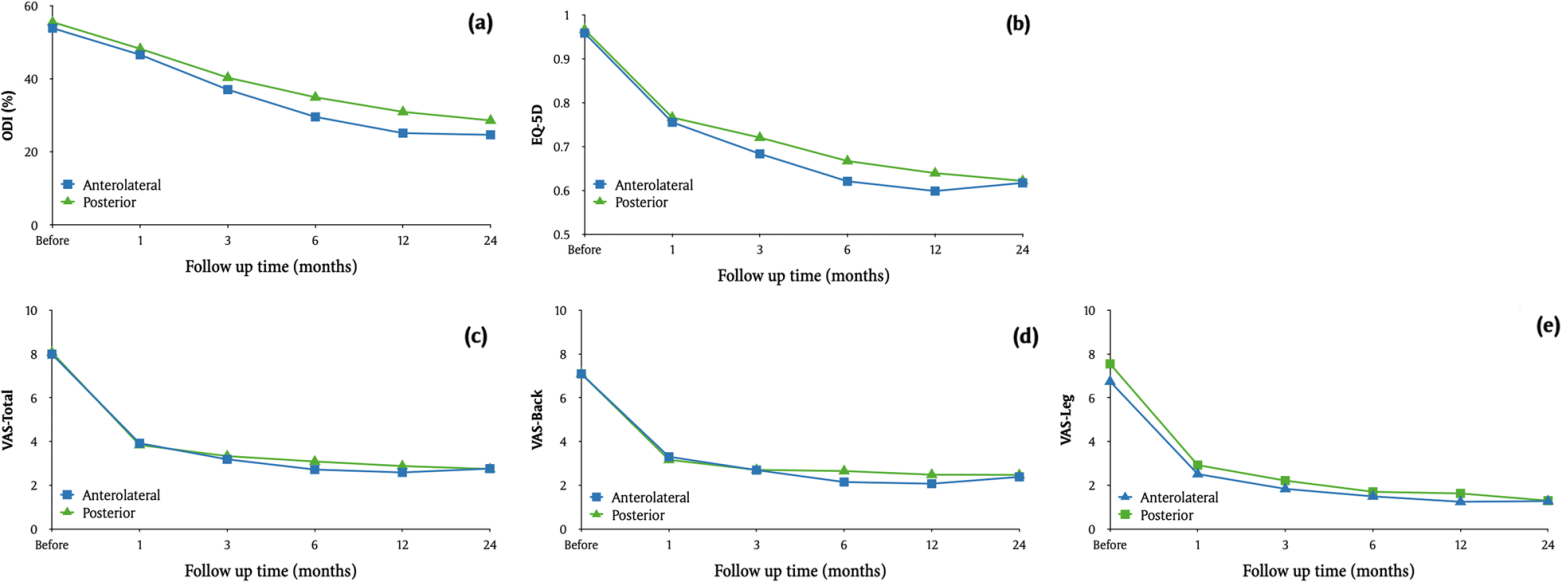
Comparison of clinical outcome between anterolateral and posterior approach over 2 years. **a** ODI **b** EQ-5D **c** VAS-Total **d** VAS-Back **e** VAS-Leg. *ALIF* Anterior lumbar interbody fusion; *OLIF* Oblique lumbar interbody fusion; *TLIF* Transforaminal lumbar interbody fusion; *ODI* Oswestry disability index; *EQ-5D* EuroQol-5-dimension score; *VAS-Total* VAS of pain in total; *VAS-Leg* VAS of pain in leg; *VAS-Back* VAS of pain in back

The comparison of HRQoL data is demonstrated in Table 4 and Fig. 3. All HRQoLs, including ODI, EQ-5D, VAS-Total, VAS-Back, and VAS-Leg, substantially improved at 2-year follow-up in the three groups, with achievements of clinical and statistical significance (*p* < 0.01). Additionally, ODI and EQ-5D in the ALIF group were superior when compared to the TLIF group at since the first month after surgery. The comparison of VAS of pain revealed no statistical significance across all approaches. The anterolateral group revealed a superior ODI and EQ-5D since the third month after surgery (Table 5 and Fig. 4). The hospital length of stay and age both negatively correlated with HRQoLs (Table 6). A higher baseline BMI showed significant relationship toward subsidence occurrence (*p* < 0.01). Patients in the TLIF group had greater achievement of the MCID in the VAS-Total than those in the ALIF and OLIF group (79.8% vs. 71% vs. 63.4%, *p* = 0.01) (Table 7).

**Table 4:** Clinical outcome of the study population stratified by lumbar interbody fusion types

HRQoL	Overall (*n* = 348)	Group			*p* Value
		ALIF (*n* = 69)	OLIF (*n* = 101)	TLIF (*n* = 178)	
*ODI*					
Pre-OP	54.8 ± 11.9	52.1 ± 14.9	55.2 ± 9.8	55.6 ± 11.6	0.11
	(53.5–56)	(48.5–55.7)	(53.2–57.1)	(53.8–57.3)	
1 M	47.5 ± 10.2	44.8 ± 11.6	47.8 ± 9.8	48.3 ± 9.8	0.02*
	(46.4–48.5)	(42.1–47.6)	(45.9–49.8)	(46.8–49.7)	
3 M	38.8 ± 12.4	34.6 ± 13	38.8 ± 12.4	40.4 ± 11.8	< 0.01**
	(37.5–40.1)	(31.5–37.8)	(36.3–41.2)	(38.6–42.1)	
6 M	32.4 ± 15	26.5 ± 16.3	31.8 ± 14.8	35 ± 13.9	< 0.01**
	(30.8–33.9)	(22.6–30.4)	(28.8–34.7)	(32.9–37)	
1 Y	28.2 ± 16.7	22.2 ± 17.2	27.2 ± 16.8	31 ± 15.8	< 0.01**
	(26.4–29.9)	(18–26.3)	(23.9–30.6)	(28.7–33.3)	
2 Y	26.7 ± 17.8	20.8 ± 17.8	27.4 ± 18.6	28.6 ± 16.9	< 0.01**
	(24.8–28.6)	(16.5–25)	(23.7–31.1)	(26.1–31.1)	
*p* value (2 Y-pre)	< 0.01**	< 0.01**	< 0.01**	< 0.01**	
EQ-5D					
Pre-OP	0.96 ± 0.09	0.94 ± 0.13	0.97 ± 0.06	0.97 ± 0.08	0.06
	(0.95–0.97)	(0.91–0.97)	(0.96–0.98)	(0.96–0.98)	
1 M	0.76 ± 0.09	0.73 ± 0.12	0.77 ± 0.07	0.77 ± 0.08	< 0.01**
	(0.75–0.77)	(0.7–0.76)	(0.76–0.79)	(0.75–0.78)	
3 M	0.7 ± 0.13	0.66 ± 0.14	0.7 ± 0.13	0.72 ± 0.13	< 0.01**
	(0.69–0.72)	(0.63–0.69)	(0.67–0.72)	(0.7–0.74)	
6 M	0.64 ± 0.16	0.59 ± 0.17	0.65 ± 0.15	0.67 ± 0.16	< 0.01**
	(0.63–0.66)	(0.54–0.63)	(0.61–0.68)	(0.64–0.69)	
1 Y	0.62 ± 0.17	0.57 ± 0.18	0.62 ± 0.17	0.64 ± 0.17	0.02*
	(0.6–0.64)	(0.53–0.62)	(0.58–0.65)	(0.61–0.66)	
2 Y	0.62 ± 0.17	0.55 ± 0.17	0.66 ± 0.18	0.62 ± 0.17	< 0.01**
	(0.6–0.64)	(0.51–0.59)	(0.63–0.7)	(0.6–0.65)	
*p* value (2 Y-pre)	< 0.01**	< 0.01**	< 0.01**	< 0.01**	
VAS-Total					
Pre-OP	8 ± 1.5	8.2 ± 1.7	7.8 ± 1.4	8.1 ± 1.4	0.16
	(7.9–8.2)	(7.8–8.6)	(7.6–8.1)	(7.9–8.3)	
1 M	3.9 ± 1.6	3.8 ± 1.7	4 ± 1.6	3.8 ± 1.6	0.41
	(3.7–4.1)	(3.4–4.2)	(3.7–4.3)	(3.6–4.1)	
3 M	3.3 ± 1.8	3.1 ± 1.8	3.3 ± 1.8	3.3 ± 1.8	0.32
	(3.1–3.5)	(2.6–3.5)	(2.9–3.6)	(3.1–3.6)	
6 M	2.9 ± 2	2.7 ± 2	2.8 ± 1.9	3.1 ± 2	0.22
	(2.7–3.1)	(2.2–3.2)	(2.4–3.1)	(2.8–3.4)	
1 Y	2.7 ± 2.1	2.6 ± 2.2	2.6 ± 2.3	2.9 ± 2	0.42
	(2.5–3)	(2–3.1)	(2.2–3.1)	(2.6–3.2)	
2 Y	2.8 ± 2.3	2.4 ± 2.2	3 ± 2.5	2.7 ± 2.2	0.34
	(2.5–3)	(1.9–2.9)	(2.5–3.5)	(2.4–3.1)	
*p* value (2 Y-pre)	< 0.01**	< 0.01**	< 0.01**	< 0.01**	
VAS-Back					
Pre-OP	7.1 ± 2.7	7.4 ± 2.6	6.9 ± 2.8	7.1 ± 2.6	0.47
	(6.8–7.4)	(6.8–8)	(6.3–7.4)	(6.7–7.5)	
1 M	3.2 ± 1.9	3 ± 1.9	3.5 ± 2	3.2 ± 1.8	0.11
	(3–3.4)	(2.6–3.5)	(3.1–3.9)	(2.9–3.4)	
3 M	2.7 ± 1.8	2.7 ± 2	2.7 ± 1.6	2.7 ± 1.9	0.89
	(2.5–2.9)	(2.3–3.2)	(2.3–3)	(2.4–3)	
6 M	2.4 ± 2	2.1 ± 2	2.2 ± 1.7	2.7 ± 2.1	0.17
	(2.2–2.6)	(1.6–2.6)	(1.9–2.5)	(2.3–3)	
1 Y	2.3 ± 2	2.1 ± 2	2.1 ± 2	2.5 ± 2	0.25
	(2.1–2.5)	(1.6–2.6)	(1.7–2.5)	(2.2–2.8)	
2 Y	2.4 ± 2.3	2.1 ± 2.1	2.6 ± 2.4	2.5 ± 2.3	0.42
	(2.2–2.7)	(1.6–2.6)	(2.1–3.1)	(2.1–2.8)	
*p* value (2 Y-pre)	< 0.01**	< 0.01**	< 0.01**	< 0.01**	
VAS-Leg					
Pre-OP	7.2 ± 2.7	6.7 ± 3	6.8 ± 3	7.5 ± 2.4	0.08
	(6.9–7.4)	(6–7.4)	(6.2–7.4)	(7.2–7.9)	
1 M	2.7 ± 2.4	2.6 ± 2.4	2.5 ± 2.4	2.9 ± 2.4	0.29
	(2.5–3)	(2–3.2)	(2–2.9)	(2.6–3.3)	
3 M	2 ± 2.3	1.8 ± 2.4	1.8 ± 2.4	2.2 ± 2.2	0.16
	(1.8–2.3)	(1.3–2.4)	(1.4–2.3)	(1.9–2.5)	
6 M	1.6 ± 2.2	1.6 ± 2.2	1.4 ± 2.2	1.7 ± 2.2	0.62
	(1.4–1.8)	(1.1–2.2)	(1–1.8)	(1.4–2)	
1 Y	1.4 ± 2.3	1.3 ± 2.1	1.2 ± 2.4	1.6 ± 2.3	0.33
	(1.2–1.7)	(0.8–1.8)	(0.8–1.7)	(1.3–2)	
2 Y	1.3 ± 2.3	1.3 ± 2.2	1.2 ± 2.6	1.3 ± 2.2	0.55
	(1–1.5)	(0.8–1.9)	(0.7–1.8)	(1–1.6)	
*p* value (2 Y-pre)	< 0.01**	< 0.01**	< 0.01**	< 0.01**	

*p* value < 0.05 was consider statistically significant. Values expressed as the mean ± standard deviation and 95 confidence interval range in the brackets. *p* < 0.05*, *p* < 0.01**

*ALIF* Anterior lumbar interbody fusion, *OLIF* Oblique lumbar interbody fusion, *TLIF* Transforaminal lumbar interbody fusion, *HRQoL* Health-related quality of life, *ODI* Oswestry disability index, *EQ-5D* EuroQol-5-dimension score, *VAS-Total* VAS of pain in total, *VAS-Leg* VAS of pain in leg, *VAS-Back* VAS of pain in back, *M* Month, *Y* Year

**Table 5:** Clinical outcome of the study population stratified by lumbar interbody fusion types

HRQoL	Group		*p* value
	Anterolateral (A/OLIF, *n* = 170)	Posterior (TLIF, *n* = 178)	
*ODI*			
Pre-OP	53.9 ± 12.2	55.6 ± 11.6	0.1
	(52.1–55.7)	(53.8–57.3)	
1 M	46.6 ± 10.6	48.3 ± 9.8	0.04*
	(45–48.2)	(46.8–49.7)	
3 M	37.1 ± 12.8	40.4 ± 11.8	< 0.01**
	(35.1–39)	(38.6–42.1)	
6 M	29.6 ± 15.6	35 ± 13.9	< 0.01**
	(27.2–32)	(32.9–37)	
1 Y	25.2 ± 17.1	31 ± 15.8	< 0.01**
	(22.6–27.8)	(28.7–33.3)	
2 Y	24.7 ± 18.5	28.6 ± 16.9	0.02*
	(21.9–27.5)	(26.1–31.1)	
*p* value (2 Y-pre)	< 0.01**	< 0.01**	
*EQ-5D*			
Pre-OP	0.96 ± 0.09	0.97 ± 0.08	0.18
	(0.94–0.97)	(0.96–0.98)	
1 M	0.76 ± 0.09	0.77 ± 0.08	0.19
	(0.74–0.77)	(0.75–0.78)	
3 M	0.68 ± 0.13	0.72 ± 0.13	< 0.01**
	(0.66–0.7)	(0.7–0.74)	
6 M	0.62 ± 0.16	0.67 ± 0.16	< 0.01**
	(0.6–0.65)	(0.64–0.69)	
1 Y	0.6 ± 0.18	0.64 ± 0.17	0.01*
	(0.57–0.63)	(0.61–0.66)	
2 Y	0.62 ± 0.18	0.62 ± 0.17	0.85
	(0.59–0.64)	(0.6–0.65)	
*p* value (2 Y-pre)	< 0.01**	< 0.01**	
*VAS-Total*			
Pre-OP	8 ± 1.6	8.1 ± 1.4	0.88
	(7.8–8.2)	(7.9–8.3)	
1 M	3.9 ± 1.6	3.8 ± 1.6	0.77
	(3.7–4.2)	(3.6–4.1)	
3 M	3.2 ± 1.8	3.3 ± 1.8	0.17
	(2.9–3.5)	(3.1–3.6)	
6 M	2.7 ± 2	3.1 ± 2	0.13
	(2.4–3)	(2.8–3.4)	
1 Y	2.6 ± 2.3	2.9 ± 2	0.18
	(2.2–2.9)	(2.6–3.2)	
2 Y	2.8 ± 2.4	2.7 ± 2.2	0.8
	(2.4–3.1)	(2.4–3.1)	
*p* value (2 Y-pre)	< 0.01**	< 0.01**	
*VAS-Back*			
Pre-OP	7.1 ± 2.7	7.1 ± 2.6	0.81
	(6.7–7.5)	(6.7–7.5)	
1 M	3.3 ± 2	3.2 ± 1.8	0.65
	(3–3.6)	(2.9–3.4)	
3 M	2.7 ± 1.8	2.7 ± 1.9	0.7
	(2.4–3)	(2.4–3)	
6 M	2.2 ± 1.8	2.7 ± 2.1	0.04
	(1.9–2.4)	(2.3–3)	
1 Y	2.1 ± 2	2.5 ± 2	0.07
	(1.8–2.4)	(2.2–2.8)	
2 Y	2.4 ± 2.3	2.5 ± 2.3	0.6
	(2–2.7)	(2.1–2.8)	
*p* value (2 Y-pre)	< 0.01**	< 0.01**	
*VAS-Leg*			
Pre-OP	6.7 ± 3	7.5 ± 2.4	0.05
	(6.3–7.2)	(7.2–7.9)	
1 M	2.5 ± 2.4	2.9 ± 2.4	0.1
	(2.2–2.9)	(2.6–3.3)	
3 M	1.8 ± 2.4	2.2 ± 2.2	0.06
	(1.5–2.2)	(1.9–2.5)	
6 M	1.5 ± 2.2	1.7 ± 2.2	0.26
	(1.2–1.8)	(1.4–2)	
1 Y	1.3 ± 2.3	1.6 ± 2.3	0.04
	(0.9–1.6)	(1.3–2)	
2 Y	1.3 ± 2.4	1.3 ± 2.2	0.63
	(0.9–1.6)	(1–1.6)	
*p* value (2 Y-pre)	< 0.01**	< 0.01**	

*p* value < 0.05 was consider statistically significant. Values expressed as the mean ± standard deviation and 95 confidence interval range in the brackets. *p* < 0.05*, *p* < 0.01**

*ALIF* Anterior lumbar interbody fusion, *OLIF* Oblique lumbar interbody fusion, *TLIF* Transforaminal lumbar interbody fusion, *HRQoL* Health-related quality of life, *ODI* Oswestry disability index, *EQ-5D* EuroQol-5-dimension score, *VAS-Total* VAS of pain in total, *VAS-Leg* VAS of pain in leg, *VAS-Back* VAS of pain in back, *M* Month, *Y* Year

**Table 6:** Correlation analysis between baseline characteristic and health-related quality of life obtained at 2-year follow-up

	HRQoL at 2 Years after Surgery				
Characteristic	ODI	EQ-5D	VAS-Total	VAS-Back	VAS-Leg
Age	0.24	0.16	0.11	0.11	0.08
*p* Value	< 0.01**	< 0.01**	< 0.01**	0.04*	0.15
HLoS	0.22	0.15	0.20	0.20	0.17
*p* Value	< 0.01**	< 0.01**	< 0.01**	< 0.01**	< 0.01**
EBL	0.05	0.05	0.02	0.00	0.01
*p* Value	0.34	0.40	0.93	0.95	0.88
BMI	0.07	0.08	0.01	0.06	0.00
*p* Value	0.20	0.16	0.91	0.30	0.97
OPD	0.11	0.13	0.05	0.07	0.07
*p* Value	0.04*	0.01*	0.34	0.17	0.18

Value expressed as Pearson correlation. *p* value < 0.05 was consider statistically significant. *p* < 0.05*, *p* < 0.01**

*HRQoL* Health-related quality of life, *HLoS* Hospital length of stay, *EBL* Estimated blood loss, *BMI* Body mass index, *OPD* Operative duration, *ODI* Oswestry disability index, *EQ-5D* EuroQol-5-dimension score, *VAS-Total* VAS of pain in total, *VAS-Leg* VAS of pain in leg, *VAS-Back* VAS of pain in back

**Table 7:** MCID achievement among surgical groups

HRQoL measurement	Overall (%)	Group (%)			*p* value
		ALIF	OLIF	TLIF	
*VAS*					
Total	73.3	71.0	63.4	79.8	0.01*
Back	77.0	82.6	72.3	77.5	0.28
Leg	76.4	73.9	72.3	79.8	0.31
EQ-5D	59.5	72.5	56.4	56.2	0.05
ODI	77.9	82.6	77.2	76.4	0.56

*p* value < 0.05 was consider statistically significant. *p* < 0.05*, *p* < 0.01**

*HRQoL* Health-related quality of life, *ALIF* Anterior lumbar interbody fusion, *OLIF* Oblique lumbar interbody fusion, *TLIF* Transforaminal lumbar interbody fusion, *ODI* Oswestry disability index, *EQ-5D* EuroQol-5-dimension score, *VAS-Total* VAS of pain in total, *VAS-Leg* VAS of pain in leg, *VAS-Back* VAS of pain in back

## Discussion

In this study, the anterolateral approach group (A/OLIF) had a lower subsidence rate, superior sagittal alignment profiles, and better clinical outcomes when compared to the posterior approach method (TLIF). The ALIF group demonstrated the best alignment correction and clinical outcome; however, less than 20% of the surgical levels were above L3. The OLIF group had the advantage of reducing blood loss, restoring sagittal alignment profiles, and being accessible at all lumbar levels while achieving comparable clinical improvement when compared to TLIF. The age and length of hospital stay were negatively correlated with clinical outcome. To the best of our knowledge, this is the first study comparing the radiographic and clinical outcomes between ALIF, OLIF, and TLIF with posterior instrumentation, both combined and individually, with a complete 2-year follow-up.

The comparison between the anterolateral and posterior approach in improving lumbar alignment and perioperative characteristics has been a topic of debate among researchers. The emphasis has been on restoring sagittal alignment for surgical purposes, as failure to properly align the lumbar spine can result in iatrogenic flatback malalignment and subsequent degeneration of adjacent segments. [12]. Reports comparing ALIF, LLIF, and TLIF identified LL and disk height (DH) demonstrated greater extent in the A/LLIF when compared to TLIF, particularly in the ALIF group [5, 13–15]. Recently, the large multicenter retrospective study performed at 6 months following surgery compared anterolateral (A/LLIF) to posterior (T/PLIF) approach reported that anterolateral procedures resulted in greater SL and PI-LL mismatch improvement at L4-L5 and L5-S1 when compared to posterior approaches [16].

An alternative use of OLIF with lateral cortical screw fixation showed superior ODI and VAS-Back while comparing to minimal-invasive TLIF [17]. Researchers have suggested that the effectiveness of indirect decompression and avoidance of back muscle violation in OLIF provides patients with better postoperative recovery and reduces the risk of complications. However, they also found that performing a posterior fixation in conjunction with cage implantation demonstrated a fusion rate of more than 90%, which was significantly higher than the 65–83% seen with stand-alone cage implantation [18]. In the current study, posterior instrumentation was applied to all patients to sufficiently correct lumbar alignment and stabilize the fusion structure. This allowed for an adequate corrective maneuver for restoring lumbar lordosis. The restoration of lumbar lordosis in the OLIF and TLIF groups may have been achieved through the distraction of the posterior structure since the anterior longitudinal ligament was preserved [19]. Additionally, the release of the anterior longitudinal ligaments allows for greater extension of the anterior column. In the current study, sagittal profiles across all approaches improved and were similar at the 6-month follow-up. However, a greater deterioration in SL, LL, SS, and increased PI-LL mismatch was found in the TLIF group, leading to a worse radiographic outcome when compared to the ALIF and OLIF groups when the follow-up period was extended to 2 years. This loss of realignment may have resulted from the relatively high subsidence rate (16.1%, *p* = 0.04) in the TLIF group. Thus, with regard to previous reports, the anterolateral approach may have superior advantages for long-term lumbar and global alignment correction when compared to the posterior approach method. Therefore, the size and lordotic angle of the implant cage and the intraoperative preparation for implantation may be the main modifiers for determining the surgical outcomes of these techniques.

Expectedly, the restoration of disk height indirectly increased neuroforaminal volume and decompressed the nerve roots, which could be easier achieved through a larger cage design, such as ALIF and OLIF cages. The avoidance of a laminectomy in the anterolateral approach preserved the integrity of the posterior anatomy; thus, we revealed a significantly better ODI and EQ-5D, but similar VAS in Total, in Back, and in Leg among the anterolateral cohorts (A/OLIF) while comparing them to TLIF. However, due to anatomical concern [4], in the current study, less than 20% of the surgical levels for ALIF were above L3. The OLIF approach provides easier access for multiple levels, eliminates the need for posterior column destruction, and provides surgeons with a wider space for bony graft placement, which in turn decreases the risk of iatrogenic injury to the surrounding soft tissue. Despite the risk to the peritoneal structure with the OLIF approach, the relatively lower risk of iatrogenic damage highlights the benefits of this method. This has been reported and concluded in the current study. [20]. In our previous study, we demonstrated the comparable clinical efficacy between ALIF and OLIF with no clinical symptomatic ASD having been developed, despite the flattened shape change in the supra-adjacent disk during a 2-year follow-up period [21]. All in all, OLIF possessed advantage in reducing blood loss, restoring lumbar lordosis and the accessibility at L1 to L4 level while simultaneously achieving comparable clinical improvement.

The current study is subject to limitations. The relatively small number of study subjects may be insufficient for representation of the general population. However, with the attending physicians performing the surgeries all having been trained in the same medical center, we have provided relatively large study subject numbers, with the surgical tools and implants being restrained unitarily, which could have minimized inconsistencies. Additionally, the adoption of a surgical level may be affected by the suitability of approach. For instance, approaching segments above L4 in ALIF, vessel injuries could be of the greatest concern [4], selection bias could not be neglected thusly.

## Conclusion

In treating degenerative lumbar disorders, the ALIF approach demonstrated excellent alignment correction and clinical outcomes. The OLIF approach has the advantages of reducing blood loss, restoring sagittal profiles, and being accessible at all lumbar levels while achieving comparable clinical improvement when compared to the TLIF approach. Patient selection and surgeon preference are crucial factors to consider when choosing a surgical approach strategy.

## Data Availability

The datasets generated during and/or analyzed during the current study are available from the corresponding author on reasonable request.

## Abbreviations

HRQoL: Health-related quality of life
LIF: Lumbar interbody fusion
PLIF: Posterior lumbar interbody fusion
TLIF: Transforaminal lumbar interbody fusion
ALIF: Anterior lumbar interbody fusion
LLIF: Lateral lumbar interbody fusion
OLIF: Oblique lumbar interbody fusion
HLoS: Hospitalization length of stay
BMI: Body mass index
EBL: Estimated blood loss
OPD: Operative duration
LL: Lumbar lordosis
PI: Pelvic incidence
PT: Pelvic tilt
SS: Sacral slope
SVA: Sagittal vertical axis
ODI: Oswestry disability index
EQ-5D: EuroQol-5-dimension score
VAS-Total: Visual analogue scale of pain in total
VAS-Leg: Visual analogue scale of pain in leg
VAS-Back: Visual analogue scale of pain in back
MCID: Minimal clinically important difference
ANOVA: One-way analysis of variance
